# Dentinogenic ghost cell tumor treated with a combination of marsupialization and radical resection: a case report and review of the literature

**DOI:** 10.1186/s13256-023-03861-w

**Published:** 2023-03-30

**Authors:** Soichiro Toyodome, Tomoko Wakasa, Katsutoshi Hirose, Noriko Iwamoto, Seiya Suzuki, Naoto Nemoto, Satoru Toyosawa, Tetsuji Nagata

**Affiliations:** 1 Department of Oral and Maxillofacial Surgery, Kindai University Nara Hospital, 1248-1 Otodacho, Ikoma, Nara 630-0293 Japan; 2 Department of Diagnostic Pathology, Kindai University Nara Hospital, 1248-1 Otodacho, Ikoma, Nara 630-0293 Japan; 3 Department of Oral Pathology, Osaka University Graduate School of Dentistry, 1-8 Yamadaoka, Suita, Osaka 565-0871 Japan

**Keywords:** Dentinogenic ghost cell tumor, Calcifying odontogenic cyst, Ghost cell, Maxilla

## Abstract

**Background:**

Dentinogenic ghost cell tumor is a rare benign tumor that accounts for less than 3% of all cases and consists of the stellate reticulum, which is made up of enamel epithelioid and basaloid cells. Although DGCT is a benign tumor, the local infiltration of the odontogenic epithelium or recurrences have been reported, and its detailed pathology and treatments remain unclear.

**Case presentation:**

This report describes the case of a 60-year-old Japanese male diagnosed with a maxillary dentinogenic ghost cell tumor. Images showed well-circumscribed, multilocular cystic lesions with a calcified substance in the interior. Marsupialization was performed along with biopsy to prevent the expansion of the lesion, and a partial maxillectomy was performed 2 years after the initial examination. Histopathological findings showed ameloblastomatous proliferation containing clusters of ghost cells and dentinoid materials, resulting in the diagnosis of dentinogenic ghost cell tumor. This article also reviews recently reported cases of dentinogenic ghost cell tumor.

**Conclusion:**

It is important to perform marsupialization, proper resection, and postoperative follow-up because of possible recurrence.

## Background

Dentinogenic ghost cell tumors (DGCT) are composed of stellate reticulum consisting of enamel epithelioid and basaloid cells; these tumors also include ghost cells with multiple characteristics and osteodentines. Although DGCT is a benign tumor, the local infiltration of the odontogenic epithelium has been observed [1]. DGCT was previously considered a solid variant of calcifying odontogenic cyst (COC); however, in 2005, the World Health Organization (WHO) reclassified COC into two subtypes: calcifying cystic odontogenic tumor (CCOT), which presents a cystic morphology, and the solid DGCT [2]. Since 2017, the WHO has continued to classify DGCT as a tumor. However, they reclassified CCOT as an odontogenic cyst and renamed it COC [3, 4]. Surgical treatments, such as curettage or resection, are the primary treatment modalities for this condition; however, recurrences have been reported, and the detailed pathology and treatments remain unclear.

In this study, we achieved good outcomes by combining marsupialization and radical resection for a case of DGCT that developed on the maxilla and report herein with a review of previous cases reported as DGCT.

## Case presentation

A 60-year-old Japanese man presented at our hospital in April 2018. The patient, who had a medical history of angina pectoris and no family history, complained of protrusion of the left maxilla and a sensation of nasal obstruction.

The patient had noticed a bulge in the left maxillary gingiva for several years before the first examination. However, the bulge was ignored, as it was not painful. Although the patient’s face deformed gradually, the patient did not undergo treatment for financial reasons. Thus, the patient developed a sensation of nasal obstruction that started several months before the first examination and then visited our department.

**Fig. 1:**
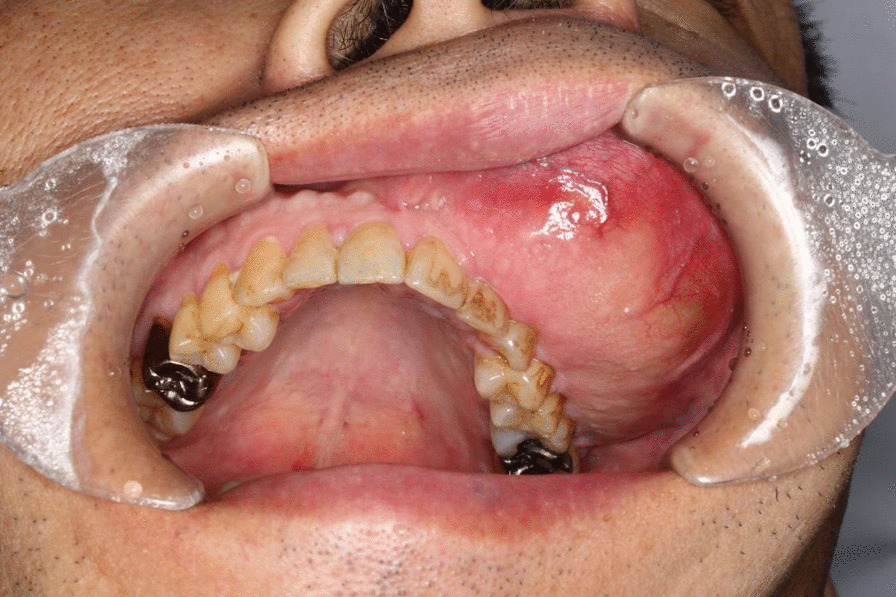
Intraoral findings at the time of first examination. Bulging was observed from the labial and buccal gingiva to buccal mucosa corresponding to the left upper central incisor to second molar

For extraoral findings, the facial asymmetry was due to bulging of the left maxillary body from the left nasal ala to the left upper lip. No hypoesthesia was observed of the infraorbital region. For intraoral findings, an approximately 60 × 40 × 30-mm well-circumscribed elongated bone-like bulge with a flat and smooth surface was observed from the labial and buccal gingiva to the buccal mucosa corresponding to the left upper central incisor of the second molar (Fig. 1). No vital response was observed on the first molar to the left maxillary canine; both were observed with mobility.

**Fig. 2:**
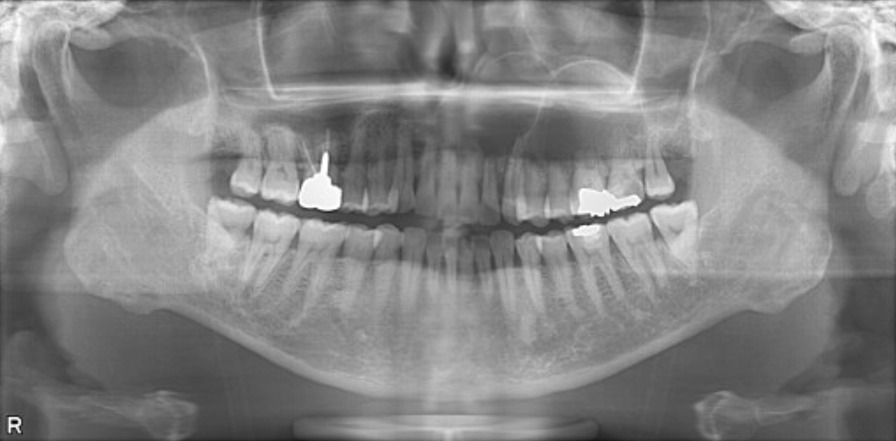
Panoramic X-ray findings at the initial examination. A multilocular cystoid radiolucency was observed around the left maxillary sinus, and knife-cut-like root resorption was noted

**Fig. 3:**
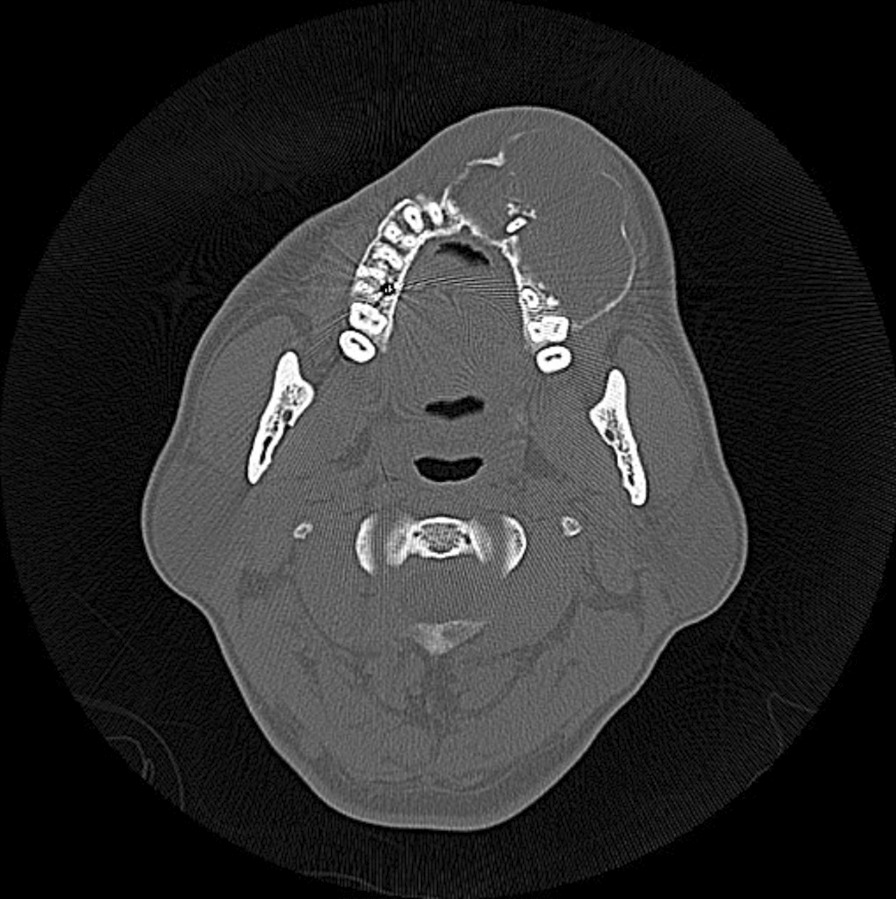
Computed tomography findings at the initial examination. A multilocular cyst-like lesion was observed in the right maxilla, and interspersed radiopacity in the interior of the solid lesion

The first panoramic X-ray image showed radiolucent multilocular cystoid images around the left maxillary sinus and the disappearance of the border of the maxillary sinus. Knife-cut-like resorption of the dental roots of the maxillary teeth extending from the right central incisor to the left second molar was noted (Fig. 2). Computed tomography (CT) images showed that a well-circumscribed, radiolucent, multilocular cyst-like 60 × 50 × 40-mm lesion, extending from the right maxillary central incisor to the left maxillary second molar and from the alveolar ridge to the floor of the nasal cavity and maxillary sinus, was observed. The interior of the tumor was solid and had partial radiopaque areas with calcification that were interspersed (Fig. 3). For financial reasons, the patient did not consent to a magnetic resonance imaging (MRI) examination; hence, it was not performed.

**Fig. 4:**
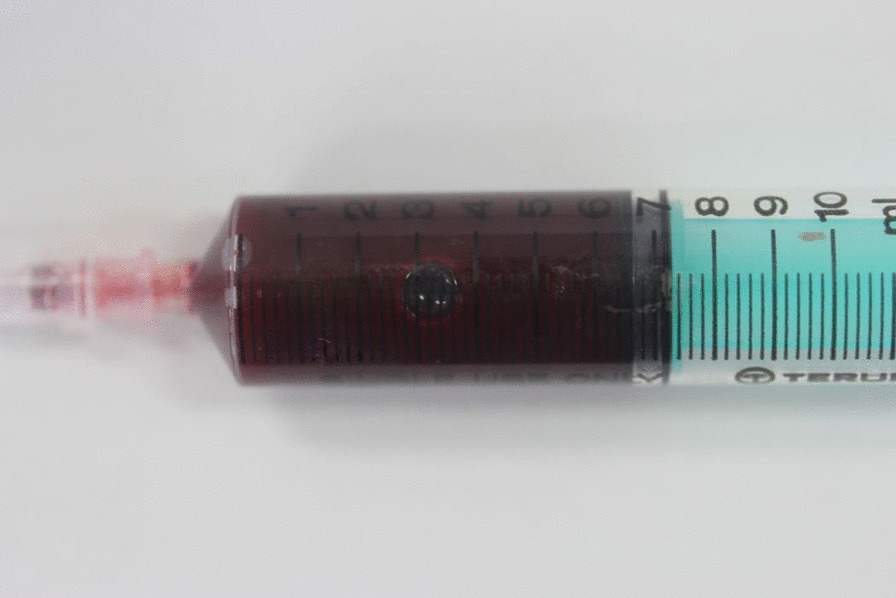
Aspirated fluid. A dark red serous fluid was aspirated from the interior of the lesion

**Fig. 5:**
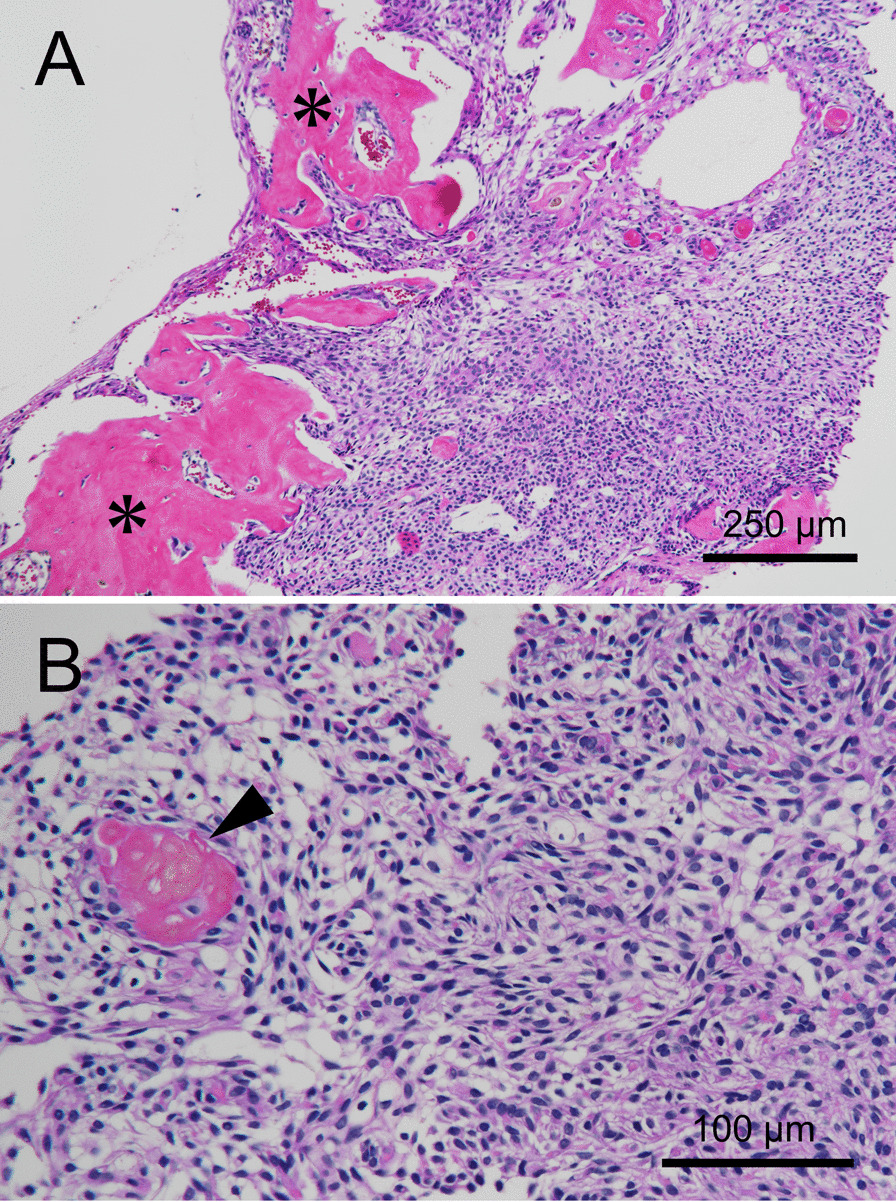
Histopathological findings of the biopsied specimen. **A** The proliferation of neoplastic epithelial cells resembling the stellate reticulum and the formation of dentinoid tissues were observed (Hematoxylin Eosin staining scale bar 250 μm) (asterisks indicate dentinoid tissues). **B** A few ghost cells were observed in the tumor nests (Hematoxylin Eosin staining scale bar 100 μm) (arrowhead indicates ghost cells)

Following these findings, we made a clinical diagnosis of a left maxillary bone neoplasm and considered the treatment course. Clinically, the progression of the tumor was suspected and indications for surgery were explained to the patient. However, the patient did not consent to surgery as an initial treatment for financial reasons and the need to continue providing long-term care for his ill wife. Concurrently, the patient complained of a sensation of left nasal obstruction, and, to avoid further progression of the tumor, marsupialization and biopsy were performed. Under local anesthesia, a fine needle aspiration was performed and a dark red serous fluid was obtained (Fig. 4). A 30 × 10-mm incision was made in the central part of the lesion, revealing the thinned bone and a thick soft tissue lesion inside it. Thus, it was peeled from the surrounding bone to sample the specimen. At this time, the same liquid that was aspirated earlier was found in the lesion, but no calcified substance was observed. The soft tissue inside the lesion and oromucosal epithelium was sutured with absorbable sutures and marsupialized. Histological examination revealed the proliferation of neoplastic epithelial cells resembling the stellate reticulum of the enamel organ (Fig. 5A). Dentinoid tissues were formed adjacent to the neoplastic epithelial cells (asterisks in Fig. 5A). Furthermore, a few ghost cells were observed in the tumor nests (Fig. 5B). The tumor cells showed no nuclei atypia and few mitotic figures. On the basis of these histological findings, benign odontogenic tumors, including odontoma, ameloblastic fibro-odontoma, COC, and DGCT, were suspected.

**Fig. 6:**
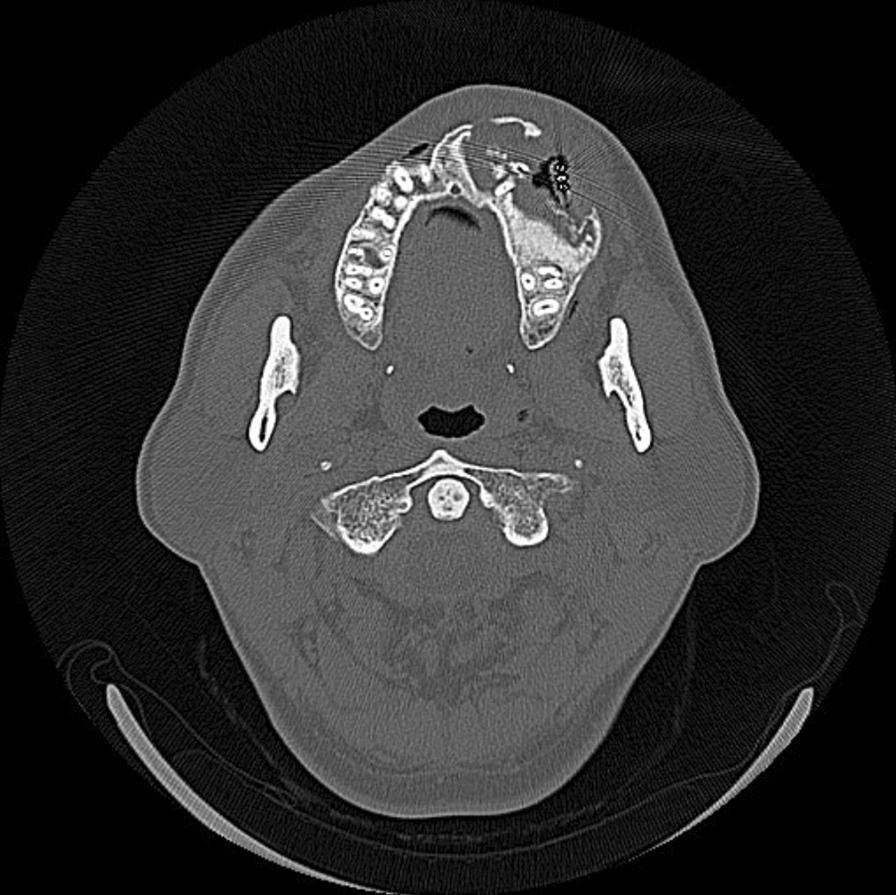
Computed tomography findings 1.5 years after marsupialization. Growth of radiopaque bone-like tissue was observed in the interior of the tumor, without metastases to the surrounding tissues

After marsupialization, the patient was fitted with an obturator and buccal protrusion gradually decreased. CT scans performed in December 2018 and 2019 showed the growth of radiopaque bone-like tissue in the interior of the tumor without metastases to the surrounding tissues (Fig. 6). As the nasal obstruction reduced, the patient became aware of symptomatic improvement and consented to surgery during follow-up in December 2019.

**Fig. 7:**
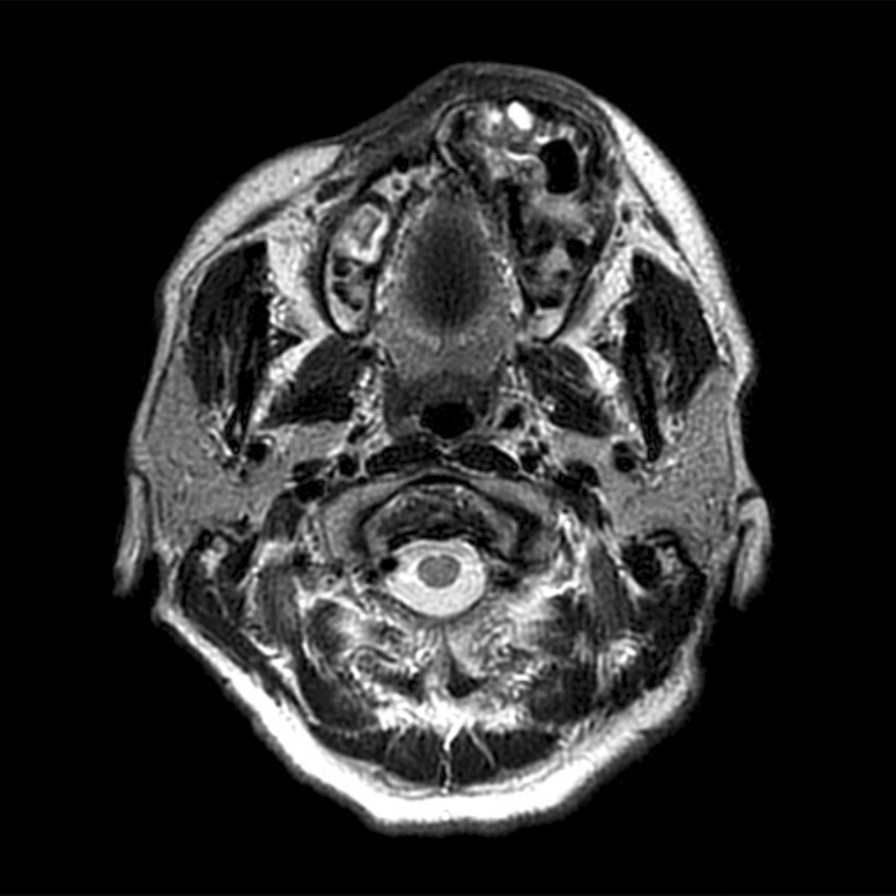
Magnetic resonance imaging findings immediately before surgery. Lesion with mixed low- and high-signals observed from the left alveolar ridge to left maxillary sinus on T2-weighted imaging was observed, without clear metastases to the surrounding tissues

To confirm the absence of new lesions in the surrounding tissues, an MRI was performed immediately before surgery. Lesions with mixed low- and high-signals observed from the left alveolar ridge to the left maxillary sinus on T2-weighted imaging were observed, but no clear metastases in the surrounding tissues (Fig. 7). Under the clinical diagnosis of left maxillary bone neoplasm, a partial left maxillectomy and reconstruction with a split-thickness skin graft were performed in June 2020 under general anesthesia.

**Fig. 8:**
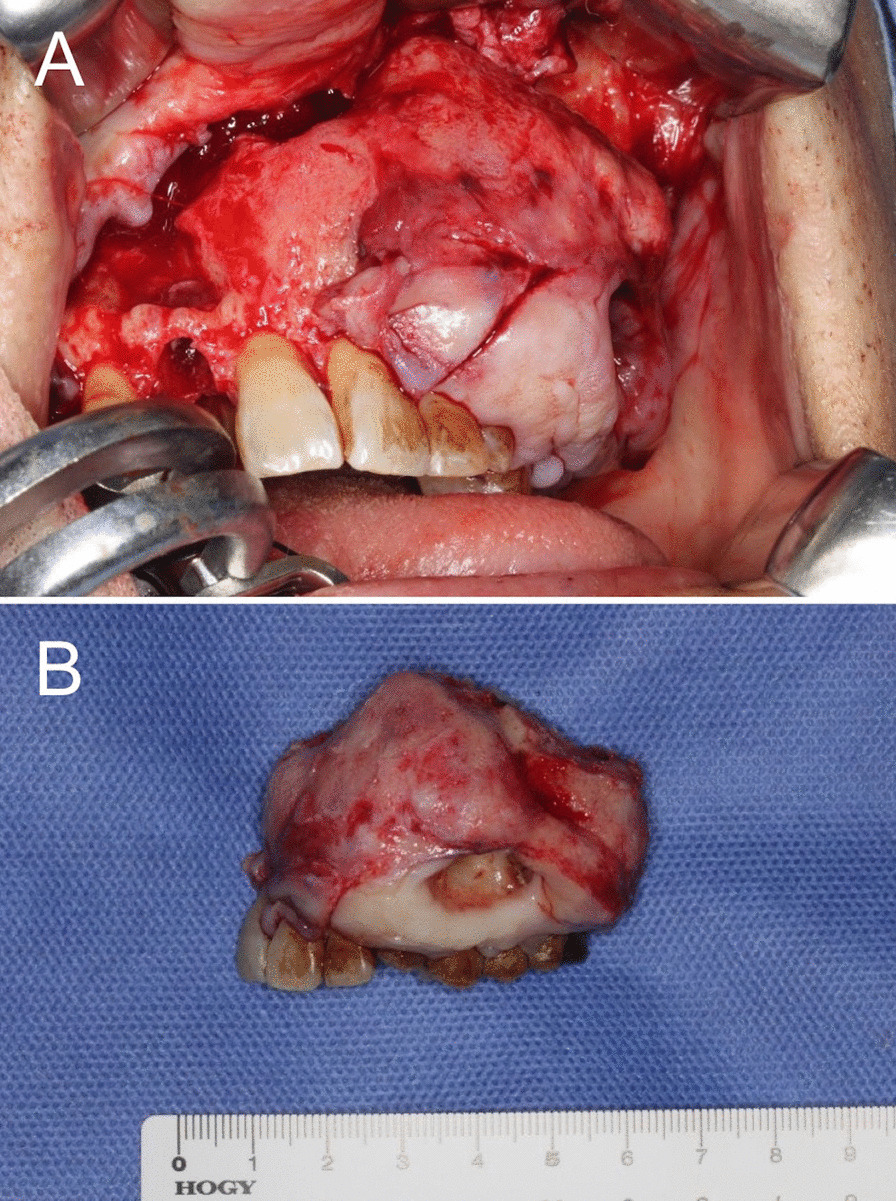
Intraoral findings at the time of surgery and photograph of the excised specimen. **A** The resection was made from the right upper incisors on the anterior side to the left upper first molar in the posterior side, and from the center of the maxillary sinus on the superior side to the floor and lateral wall of the nasal cavity on the medial side. **B** The interior of the lesion was solid. The resected specimen includes surrounding tooth and bone tissues

**Fig. 9:**
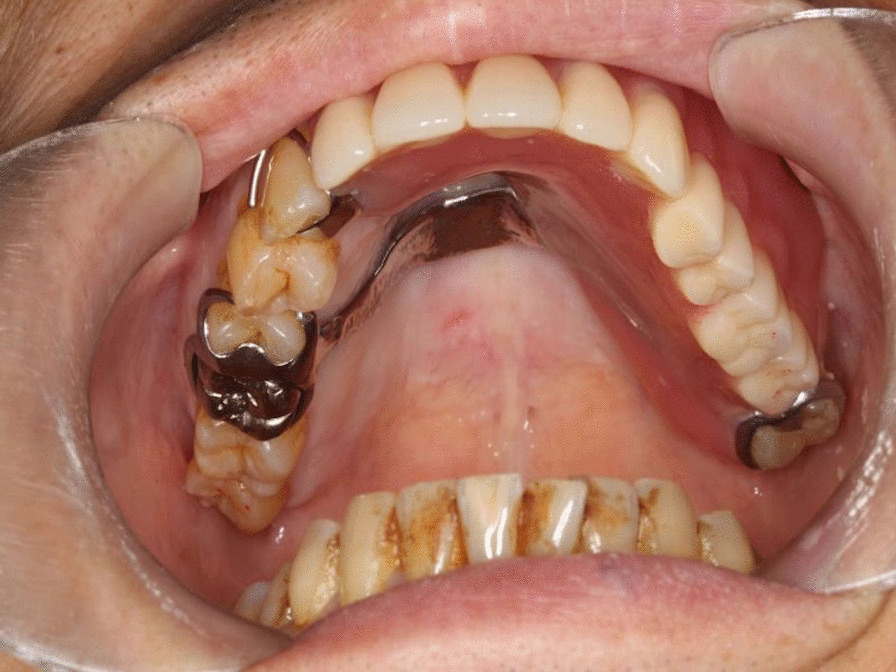
Intraoral photograph 1 year after surgery. No clear recurrence was observed. The patient wears a maxillary prosthesis and is in stable condition, both functionally and esthetically

A Weber-Ferguson incision was made from the left filtrum to the nasal ala to secure the operative field. The buccal aspects of the left maxilla and the tumor were exposed, and radical resection with a safety margin of approximately ≥ 5 mm was planned. The oral mucosa was cut to include tumor marsupialization (Fig. 8a). Partial maxillectomy was performed from the right upper incisor to the left upper first molar, including the bottom of the maxillary sinus. The interior of the lesion was solid (Fig. 8b). Subsequently, the raw surface of the buccal mucosa was covered with a split-thickness skin graft, filled with gauze containing tetracycline hydrochloride ointment, and protected with an acrylic splint. The postoperative course was uneventful, and a maxillary prosthesis for the defect was made in the fourth postoperative month (Fig. 9). Over 1 year has passed since the surgery and the patient has been followed up with regularly, without finding a recurrence.

**Fig. 10:**
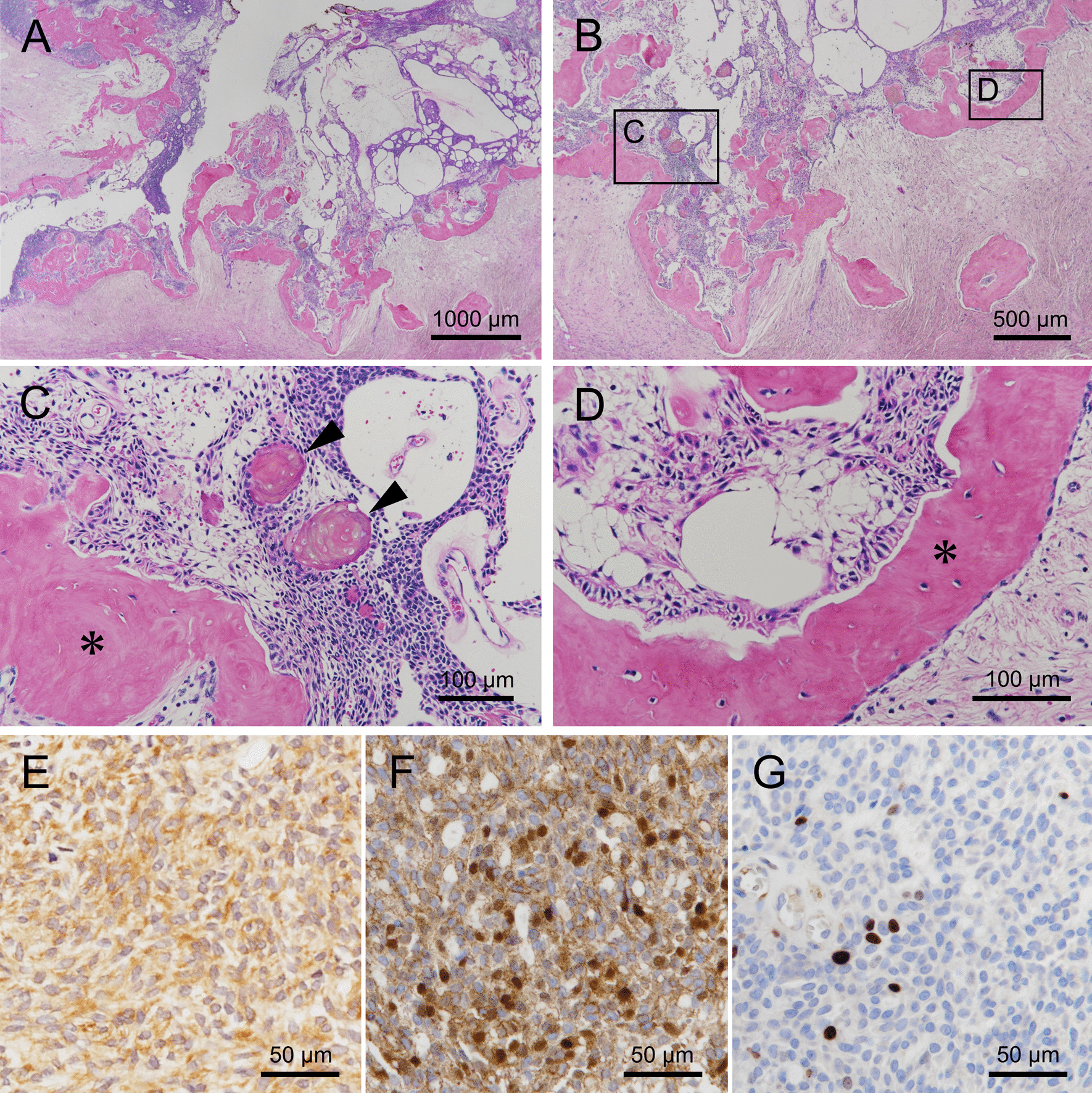
Histopathological findings of the excised specimen. **A**, **B** A large amount of dentinoid tissue and proliferation of tumor cells in the fibrous connective tissue was observed. The tumor showed solid growth, with tumor cells having a cystic morphology. (Hematoxylin Eosin staining, **A** scale bar 1000 μm, **B** scale bar 500 μm). **C** A cluster of ghost cells (arrowheads) were observed in the tumor nests containing stellate reticulum-like cells. (Hematoxylin Eosin staining, scale bar 100 μm, *: dentinoid tissue). **D** The cuboidal or columnar epithelial cells, similar to ameloblasts, presented at the periphery of the tumor nests and lined dentinoid tissues. Almost no nuclear division of tumor cells was observed. (Hematoxylin Eosin staining scale bar 100 μm, *: dentinoid tissue). **E** The cytoplasm and membrane were positive for cytokeratin AE1/AE3 (immunohistochemical staining scale bar 50 μm). **F** The nucleus and cytoplasm were positive for β-catenin (immunohistochemical staining scale bar 50 μm). **G** MIB1 (Ki67) index was < 3% (immunohistochemical staining scale bar 50 μm)

Histopathological findings: the excised specimen revealed that a collagenous fibrous capsule encapsulated the lesion, and no invasive proliferation was observed in the adjacent tissues (Fig. 10A, B). The tumor was composed mainly of stellate reticulum-like cells with large areas of dentinoid formation (Fig. 10C). The cuboidal or columnar epithelial cells, similar to the ameloblasts, were presented at the periphery of the tumor nests and lined dentinoid tissues (Fig. 10D). A cluster of ghost cells was observed in the tumor nests (arrowheads in Fig. 10C). Vascular invasion, perineural invasion, or necrosis were not seen. Immunohistochemistry showed that the tumor cells were positive for cytokeratin AE1/AE3 (Fig. 10E), while nuclear and cytoplasmic were positive for β-catenin (Fig. 10F). The MIB1 (Ki67) index was < 3% (Fig. 10G). We then made a final diagnosis of DGCT.

## Discussion

The term DGCT was first proposed by Praetorius *et al*. in 1981. They suggested this name because the formation of dentinoid in relation to the epithelial islands was a conspicuous feature and ghost cells were found to a varying degrees [5]. Later, various other names were used to refer to the rare variants of solid calcifying odontogenic cysts, but since the 2005 WHO classifications, they have become clearly distinguished as a lesion with characteristics of a tumor, of which the cystic varieties were termed CCOT and the solid version as DGCT [6]. Furthermore, the 2017 WHO classifications classified DGCT as a tumor, and CCOT became considered an odontogenic cyst and was renamed COC. The WHO defines DGCT as a benign tumor composed of dispersed enamel epitheliomatous cells and basaloid and stellate reticulum cells with local invasion containing ghost cells and aberrant keratinization. According to the WHO, the incidence is less than 3%, and more than half of reported cases were in Asian patients. The male-to-female ratio is 2:1, and it is most frequent in patients in from 40–60 years old. Intraosseous and peripheral types of DGCT exist, and the molar regions of the mandible and maxilla are preferred sites [4]. To date, Juneja *et al*. [6] summarized the reports of intraosseous DGCT dating from 1972 to 2008, and Konstantakis [7] summarized the cases reported in the subsequent period to 2013 in a review. Thus, we searched for reports related on DGCT written in English between 2014 and 2020 on PubMed (Table 1). Similar to past reviews, cases with malignant transformation or peripheral types were excluded. Moreover, we also excluded articles in which the DGCT diagnosis or its localization was unclear. Zhang *et al*. [8] compared these with DGCT data in their genetic analysis of odontogenic keratocysts and epithelioma, but their study was also excluded owing to a lack of clinical findings. Ahire *et al*. [9] investigated odontogenic tumors in 250 cases over the past 35 years, but their investigation was based on the 2017 WHO classifications and was thus included in this review.

**Table 1:** List of articles reporting intraosseous dentinogenic ghost cell tumor in 2015–2020

Author and year	Cases	Age, year	Sex	Site	Radiographic findings	Treatment	Follow up
Urs *et al*., 2020	7	9	M	Mandible	Radiolucent	NA	NA
		26	F	Mandible	Radiolucent	NA	NA
		15	M	Mandible	Radiolucent	NA	NA
		18	F	Maxilla	Radiolucent	NA	NA
		21	M	Mandible	Radiolucent	NA	NA
		60	M	Mandible	Radiolucent	NA	NA
		15	F	Mandible	Radiolucent	NA	NA
Zhang *et al*., 2020	6	NA	NA	NA	NA	NA	NA
Ravi *et al*., 2020	1	12	F	Left side mandible	Multiple, well defined, irregular radiopacities	Completely excised	No evidence of recurrence, 2 year postoperatively
Bavle *et al*., 2020	2	28	M	Left maxillary quadrant	Radiopaque mass	Conservative surgical removal	Recurrent lesion found after 11 months
		21	F	Lower left molar	Well-demarcated radiolucency with multiple specks of radiopacities	Conservative excision	No complaints of recurrence after 3 years of follow-up
Patankar *et al*., 2019	1	18	M	Maxillary left region	CT scan showed a mixed hypodense hyperdense lesion	Segmental resection	No recurrence after 1 year of surgery
Gupta *et al*., 2019	1	40	M	Right maxilla	NA	Excised under local anesthesia	NA
Bussari *et al*., 2019	1	40	F	Mandible and extending into the submental region	Orthopantomograph revealed a multilocular radiolucency	Segmental mandibulectomy	No recurrence after 9 months of surgery
Rosa *et al*., 2019	5	31	F	NA	NA	NA	NA
		71	M	Mandible	Radiolucent	NA	NA
		NA	M	Maxilla	Radiolucent	NA	NA
		42	F	Mandible	Radiolucent	NA	NA
		65	F	Maxilla	NA	NA	NA
Korranne *et al*., 2018	1	26	M	Left maxilla	NA	Excised under local anesthesia	NA
Soares *et al*., 2018	1	38	M	Anterior mandible and protruding extraorally	CT showed an extensive exophytic mass	Partial mandibulectomy	NA
Ahire *et al*., 2018	1	50s	M	Mandible	NA	NA	NA
Sheikh *et al*., 2017	1	65	F	Left mandibular body	Mixed radiopaque and radiolucent mass	Excision with peripheral ostectomy	No recurrence after 4 months of surgery
Walia *et al*., 2017	1	80	F	Right maxillary alveolar ridge	Occipitomental radiograph showing mixed radiopaque and radiolucent lesion	En bloc resection	No recurrence after 6 months of surgery
Agrawal *et al*., 2017	1	14	M	Right mandibular premolar-molar region	Radiolucent lesion with radiopaque calcified flecks	Surgical enucleation	No recurrence
Kennedy *et al*., 2017	1	32	M	Left mandible	Partially solid, radiolucent lesion	Enucleation	No evidence of recurrence on clinical and radiographic review 5 months later
Bafna *et al*., 2016	1	68	M	Lower anterior region of the jaw	Radiolucent lesion	Local excision	No recurrence after 7 months of surgery
Rai *et al*., 2015	1	33	F	Left maxilla	Mixed radiolucency	Enucleation	No recurrence after 2 years of surgery

*NA* individual data are not available, *M* male, *F* female, *CT* computed tomography

The 27 cases we searched for in this review consisted of 3 retrospective studies [9–11] and 13 case reports [12–24]. We added our case to these for this investigation. Patient ages ranged from 9–80 years, with a mean age of 36.5 years, which was a similar age distribution to those reported in the past reviews [6, 7]. Although the patient in our study was a 60-year-old man, the disease had a long course and was not discovered until the tumor had grown significantly, suggesting a younger age at onset. In total, 17 of the cases were on the mandible and 10 were on the maxilla, and the site was not reported for the remaining case. Previous studies included cases, such as the present case, where DGCT arose in the maxillary alveolar region and grew toward the maxillary sinus [1, 25, 26]; 16 were men and 12 were women. No clear associations were noted between systemic diseases and DGCT. Although underlying diseases, such as type 2 diabetes mellitus and hypertension, were noted in one of the cases found in our search [22], all other patients were otherwise healthy or had no documented disease history (Table 1). Our case had angina pectoris alone, also suggesting the absence of significant links between DGCT and systemic diseases.

Many of the DGCTs were well circumscribed in imaging findings with mixed radiolucency and radiopacity, as was the case with our patient. Simultaneously, DGCT can sometimes be observed as a simple radiolucent lesion. This difference is attributed to the level of calcification of the interior of the lesion [7]. Moreover, it has been noted that DGCT is associated with root resorption [7, 27], which also paralleled the finding in our patient in whom root resorption corresponding to the lesion area was observed (Figs. 2 and 3). Several studies have also reported associated impacted teeth and odontomas [24]. However, these findings are similar to enamel epithelioma or COC, making it challenging to differentiate DGCT using diagnostic imaging alone.

Hence, the DGCT diagnosis should be made on the basis of the findings of numerous ghost cells and the presence of osteodentin and solid characteristics observed on histopathological evaluation. In this study, benign odontogenic tumors, such as ameloblastic fibrodentinoma, were suspected because the biopsy performed during marsupialization showed dentin and interstitial cyst formation. However, only part of the lesion was observed, which did not provide adequate evidence to make a definitive diagnosis (Fig. 5). Stellate reticulum-like cells, cystoid structures, osteoid formation, and ghost cells were subsequently observed in the excised specimen; furthermore, the tumor was a solid growth and it was positive for cytokeratin AE1/AE3 and β-catenin on immunohistochemistry, leading to the DGCT diagnosis (Fig. 10E, F). Our case suggests that the diagnosis of DGCT should be made after extensively analyzing the overall picture through imaging and pathological findings. Moreover, the possibility of malignant transformation has been noted in DGCT, which is also essential for diagnosis [28–33]. In this study, the lack of mitotic figures and the MIB1 (Ki67) index, which was < 3% on immunohistochemistry, ruled out the possibility of malignant transformation (Fig. 10G).

Although discussions on optimal treatment are ongoing, intraosseous DGCT has been noted to behave more aggressively than peripheral DGCT [1, 7, 27, 34]. As such, most previous studies recommend extensive resection. Insufficient resection can not only cause recurrence, but can also lead to malignant transformation [33]. In the past, distant metastasis was observed on the donor side used for reconstruction following the resection of DGCT [27]. In most of the articles reviewed in this study, the resection was extensive and clear recurrence was not observed, whereas recurrence was observed in a case where conservative surgical removal was indicated [24].

Despite that, it is possible to encounter relatively enlarged lesions as well as patients for whom resection cannot be indicated smoothly owing to a lack of patient compliance, and so on. In this study, radical resection could not be performed as an initial treatment because of the patient’s financial and personal status. Therefore, marsupialization was performed first, and no further enlargement of the lesion was observed after careful subsequent follow-up, which allowed us to perform surgical removal by partial maxillectomy 2 years after the first examination. This review only includes one case [12], making it a rare case treated with marsupialization and prolonged observation. We can state that our case is rare because it was treated with a combination of marsupialization and radical resection, while almost all DGCT cases without recurrences were treated by resection. Over 1 year has passed since the surgery with no sign of recurrence. However, the possibility of local recurrence cannot be ruled out. Therefore, it is best to ultimately perform a radical resection, and it is crucial to observe the patient cautiously and postoperatively, even when marsupialization is indicated.

## Conclusion

We reported a case of DGCT that showed a good prognosis following radical resection after marsupialization and reviewed previous reports. Although reports of DGCT are few, a comprehensive diagnosis based on clinical, imaging, and pathological findings is imperative. As the possibility of local recurrence cannot be ruled out, radical resection of the tumor is necessary, even if marsupialization is performed as the first-line of treatment, and the patient should be followed up with.

## Data Availability

Data sharing is not applicable to this article as no data sets were generated or analyzed during the current study.

## Abbreviations

DGCT: Dentinogenic ghost cell tumor
COC: Calcifying odontogenic cyst
CCOT: Calcifying cystic odontogenic tumor
